# Structural Heterogeneity of Terminal Glycans in *Campylobacter jejuni* Lipooligosaccharides

**DOI:** 10.1371/journal.pone.0040920

**Published:** 2012-07-16

**Authors:** Evgeny A. Semchenko, Christopher J. Day, Marc Moutin, Jennifer C. Wilson, Joe Tiralongo, Victoria Korolik

**Affiliations:** Institute for Glycomics, Griffith University, Gold Coast, Queensland, Australia; East Carolina University School of Medicine, United States of America

## Abstract

Lipooligosaccharides of the gastrointestinal pathogen *Campylobacter jejuni* are regarded as a major virulence factor and are implicated in the production of cross-reactive antibodies against host gangliosides, which leads to the development of autoimmune neuropathies such as Guillain-Barré and Fisher Syndromes. *C. jejuni* strains are known to produce diverse LOS structures encoded by more than 19 types of LOS biosynthesis clusters. This study demonstrates that the final *C. jejuni* LOS structure cannot always be predicted from the genetic composition of the LOS biosynthesis cluster, as determined by novel lectin array analysis of the terminal LOS glycans. The differences were shown to be partially facilitated by the differential on/off status of three genes *wlaN*, *cst* and *cj1144-45.* The on/off status of these genes was also analysed in *C. jejuni* strains grown *in vitro* and *in vivo,* isolated directly from the host animal without passaging, using immunoseparation. Importantly, *C. jejuni* strains 331, 421 and 520 encoding cluster type C were shown to produce different LOS, mimicking asialo GM_1_, asialo GM_2_ and a heterogeneous mix of gangliosides and other glycoconjugates respectively. In addition, individual *C. jejuni* colonies were shown to consistently produce heterogeneous LOS structures, irrespective of the cluster type and the status of phase variable genes. Furthermore we describe *C. jejuni* strains (351 and 375) with LOS clusters that do not match any of the previously described LOS clusters, yet are able to produce LOS with asialo GM_2_-like mimicries. The LOS biosynthesis clusters of these strains are likely to contain genes that code for LOS biosynthesis machinery previously not identified, yet capable of synthesising LOS mimicking gangliosides.

## Introduction

*Campylobacter* induced enteritis is one of the major food borne diseases with increasingly high incidence rates in the developing world [1], [2], [3]. *C. jejuni* is the most common species of *Campylobacter* to infect humans. It is well documented that the LOS of some *C. jejuni* strains structurally mimics human gangliosides, particularly those found in the peripheral nervous system [4], [5], [6]. Approximately 1/1000 patients diagnosed with *C. jejuni* infection develop Guillain-Barré (GBS) or Fisher (FS) neuropathies due to the induction of anti-ganglioside cross-reactive antibodies [7], [8], [9], [10]. *C. jejuni* strains have been documented to produce a variety of LOS structures that mimic mammalian gangliosides, including those implicated in GBS (GM_1_, GM_1b_, GD_1a_, GalNAc-GD_1a_) and FS (GQ_1b_, GT_1a_) [10], [11]. Recent studies have also demonstrated that sialylation of *C. jejuni* LOS plays a key role in the onset of both neuropathies possibly through increased activation of dendritic cells and B cell proliferation that then could lead to the synthesis of cross-reactive antibodies [8], [9]. The genes involved in LOS biosynthesis have been identified following sequencing of the *C. jejuni* NCTC 11168 genome (GS), a laboratory passaged variant of the original isolate –11168-O [12]. To date, 19 LOS biosynthesis cluster types have been identified with those belonging to the ABC group able to synthesise LOS that induces the production of cross-reactive antibodies [13]. In addition, cluster types M and R contain genes that are required for the synthesis of sialylated LOS structures, but not all have been directly implicated in the development of autoimmune neuropathies.

The differential expression of genes involved in synthesis and modification of surface molecules through single nucleotide mutations is commonly attributed to the diversity of *C. jejuni* LOS structures observed [14], [15]. Furthermore, it has been suggested that environmental factors such as temperature play an important role in the modulation of bacterial metabolism as a result of the colonisation of different hosts [16], [17], [18]. Accordingly, modification of the surface antigens by *C. jejuni* through variation of gene expression and modulation of metabolism is a unique adaptation method utilised by the bacterium to promote its survival and persistence.

This study investigated the genetic basis for the production of heterogeneous LOS structures by *C. jejuni* and the mechanisms involved. Here we show that the terminal LOS structures from six isolates of *C. jejuni*, identified using a novel lectin array technique, do not fully correspond to the genetic composition of the LOS biosynthesis clusters. Furthermore, the LOS biosynthesis clusters of two human isolates of *C. jejuni* could not be identified by standard PCR approaches, indicating the presence of LOS cluster types, not previously described in the literature. Furthermore, all isolates were passaged in a co-culture with the human colonic carcinoma cell line (CaCo-2) monolayer to determine if adherence and invasion levels can be influenced by the LOS structure or LOS biosynthesis cluster types. We also analysed the effect of culturing in host adapted conditions on the on/off status of two genes *cj1144-45* and *wlaN* that are associated with the production of variable LOS structures. The on/off status of these genes was analysed in cells isolated directly from animal caeca using immunoseparation.

## Results

### LOS Biosynthesis Cluster Type Analysis

**Figure 1:**
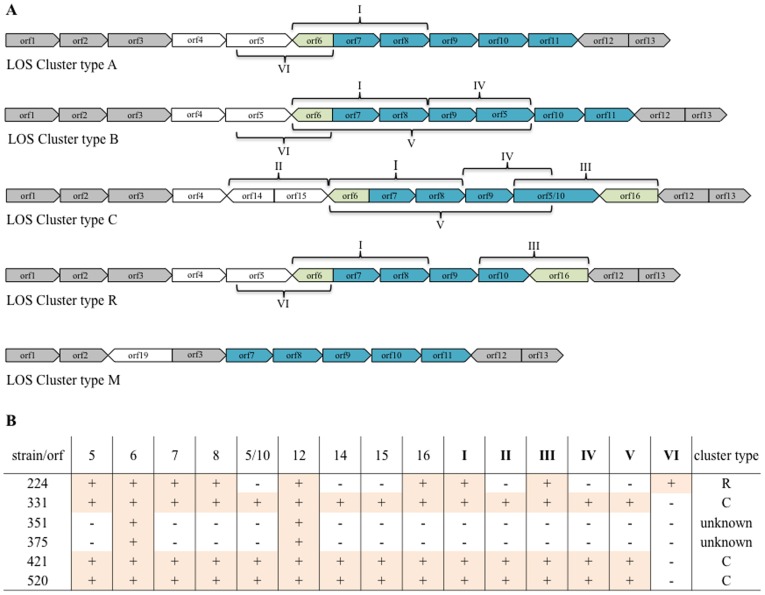
*C. jejuni* LOS biosynthesis cluster typing. A) LOS biosynthesis cluster types A, B, C, R & M and amplified DNA fragments that were used for cluster identification. B) Results of independent (orf5, orf6, orf7, orf8, orf5/10, orf12, orf14, orf15 & orf16) and overlapping (I: orf6-orf8; II: orf14–15; III: orf5/10–16; IV: orf9-orf5/10; V: orf6-orf5/10; VI orf5-orf6) PCR amplifications from the genomes of *C. jejuni* isolates 224, 331, 351, 375, 421 and 520. Plus (+) symbol denotes a positive result of the PCR amplification judged by the correct DNA fragment size and confirmed by dideoxynucleotide sequencing of the amplified DNA fragment. Minus (−) symbol denotes a negative PCR outcome, suggestive of the gene’s absence or inability to amplify it using low stringency conditions.

In order to investigate the correlation between the LOS biosynthesis cluster type and the terminal LOS structures produced by *C. jejuni* isolates, the LOS biosynthesis clusters of six uncharacterised *C. jejuni* isolates (*C. jejuni* 224, 351, 375, 421 and 520– human isolates and *C. jejuni* 331– a hyper-colonising chicken isolate) were typed using a standard PCR approach. Primer combinations for the PCR typing of the LOS gene clusters were based on the genetic organisation of *C. jejuni* NCTC 11168, the LOS biosynthesis cluster – type C (Figure 1A). Primer integrity, enabling cluster identification, was confirmed bioinformatically to have the level of sequence similarity required to amplify targeted genes from *C. jejuni* strains including those that have the LOS biosynthesis clusters A, B, C, M and R. LOS biosynthesis clusters of *C. jejuni* isolates were then typed by analysing the amplified DNA sequences of orf5 (*cgtA*), orf6 (*wlaN*), orf7 (*cstIII*), orf8 (*neuB*), orf5/10 (*cgtA/neuA*), orf12 (*waaV*), orf14 (*cj1137*), orf15 (*cj1138*) and orf16 (*cj1144-45*), and the overlapping gene fragments I: orf6-orf8 (*wlaN-neuB*), II: orf14–15 (*cj1137-cj1138*), III: orf5/10–16 (*cgtA/neuA-cj1144-45*) IV: orf9-orf5 (*neuC-cgtA*), V: orf6-orf5/10 (*wlaN-cgtA/neuA*) and VI: orf5-orf6 (*cgtA*-*wlaN*) from each of the genomes, (Figure 1B). Figure 1 shows the results of the PCR amplifications as well as the proposed LOS cluster types for *C. jejuni* isolates 224, 331, 351, 375, 421 and 520. The LOS biosynthesis cluster type could not be determined for *C. jejuni* isolates 351 and 375 as only orf12 (*waaV*) and orf6 (*wlaN*) could be amplified from their genomes despite reducing the stringency of all the amplifications and multiple optimisations, indicating that these strains may have a novel LOS cluster type.

*C. jejuni* strains 331, 421 and 520 were determined to have a LOS biosynthesis cluster type C, identical to the paradigm *C. jejuni* strain NCTC 11168-GS. *C. jejuni* 224 was deduced to have a LOS biosynthesis cluster type R by amplifying only orf6 (*wlaN*), orf7 (*cstIII*), orf8 (*neuB*), orf12 (*waaV*) and orf16 (*cj1144-45*) (Figure 1B), and the overlapping DNA fragments I: orf6-orf8 (*wlaN-neuB*) and III: orf5/10–16 (*cgtA/neuA-cj1144-45*). The amplicons were sequenced using the dideoxynucleotide approach to confirm their identity. The *in silico* analysis of *cstIII* DNA sequences from *C. jejuni* 224 showed that it has 70% and 100% sequence identity with its homologues in *C. jejuni* strains 84-25, 11168 (cluster type C) and GC149 (cluster type R) respectively. The DNA sequence analysis of *cj1144-45* from *C. jejuni* 224 showed that it has 80% and 98% sequence identity with its homologues in *C. jejuni* GC149 (cluster type R) and *C. jejuni* 11168 (cluster type C) respectively. Similarly, the DNA sequence of *wlaN* from *C. jejuni* 224 showed it has 89% identity with a partial sequence from *C. jejuni* GC149 and 98% identity with its homologue in *C. jejuni* 11168. The PCR amplified nucleotide sequence of orf7 (*cstIII* – α2,3 sialyltransferase), revealed that the gene translation is interrupted prematurely by an early stop codon (255bp) in *C. jejuni* strains 331 and 421, unlike *C. jejuni* strains 224 and 520 where the reading frame is uninterrupted. This suggests that *C. jejuni* strains 331 and 421 should not be able to sialylate their LOS. Similarly, it was shown that the *wlaN* DNA sequences were interrupted by an extra guanine residue in the polynucleotide tract in *C. jejuni* strains 421 and 520. This indicates that these strains are unlikely to produce LOS with GM_1_-like mimicries. Interestingly, the open reading frame of *cj1144-45* from *C.jejuni* 224 was shown to be uninterrupted in the polynucleotide tract, indicating that *C. jejuni* 224 has a functional *cj1144-45*.

### Host Effect on the on/off Status of the Phase Variable Genes *wlaN* and *cj1144-45*

Generation of antigenic variation by *C. jejuni* during colonisation of different hosts is generally attributed to differential expression profiles of phase variable genes. To further investigate the phenomenon of variable LOS production by *C. jejuni*, we have analysed the on/off status of two phase variable genes *cj1144-45* and *wlaN* from the LOS biosynthesis cluster following passage of *C. jejuni* isolates in different hosts. Unlike previous studies, where host adapted *C. jejuni* was analysed following an additional passage in laboratory conditions, this study investigated the on/off status of *cj1144-45* and *wlaN* genes by analysing PCR amplified full DNA sequences of these genes from the genomes of six *C. jejuni* strains (11168-O, 11168-GS, cluster type C; 224, cluster type R; 331, cluster type C; 421 cluster type C and 520, cluster type C) isolated directly from chicken caecal content and the infected CaCo-2 cells by immunoseparation. The six strains containing both *cj1144-45* and *wlaN* genes were selected for this part of the study, in order to investigate whether these genes are phase variable *in vivo* in different *C. jejuni* hosts. The baseline on/off profile of *cj1144-45* and *wlaN* genes was determined by analysing the DNA sequence of these genes amplified from the genomes of *C. jejuni* isolates passaged in laboratory conditions with the growth temperatures adjusted to mimic avian (42°C) and mammalian (37°C) hosts. As shown in Table 1, there was no change in the lengths of *cj1144-45* and *wlaN* homopolymeric tracts following growth in laboratory conditions at either 37°C or 42°C, implying that the on/off status of these genes did not change (*cj1144-45*-10A/9G tracts “on” in *C. jejuni* 224; *wlaN* –8G tract “on” in *C. jejuni* 11168-O, 11168-GS, 224 and 331).

**Table 1:** Sequence analysis of polynucleotide tracts of *cj1144-45* and *wlaN* amplified from genomes of passaged *C. jejuni* strains 11168-O, 11168-GS, 224, 331, 421 and 520.

	Polynucleotide tract lengths			
Strain	*cj1144-45*		*wlaN*	
	37°C	42°C	37°C	42°C
**11168-O**				
lab conditions	9A/9G*	9A/9G	**8G** **	**8G**
caco-2	9A/9G	–	**8G**	–
chicken	–	9A/9G	–	**8G**
**11168-GS** ****				
lab conditions	9A/8G	9A/8G	**8G**	**8G**
caco-2	9A/9G	–	**8G**	–
**224**				
lab conditions	**10A/9G**	**10A/9G**	**8G**	**8G**
caco-2	**10A/9G**	–	**8G**	–
chicken	–	10A/10G	–	9G
**331**				
lab conditions	10A/10G	10A/10G	**8G**	**8G**
caco-2	10A/10G	–	9G	–
chicken	–	10A/10G	–	9G
**421**				
lab conditions	10A/10G	10A/10G	9G	9G
caco-2	10A/10G	–	9G	–
chicken	–	***	–	9G
**520**				
lab conditions	10A/10G	10A/10G	9G	9G
caco-2	10A/10G	–	9G	
chicken	–	10A/10G	–	9G

* (Normal text) denotes the “off” status of the gene, indicating the introduction of an early stop codon due to a frame shift mutation (*cj1144-45* 10A/10G, 9A/9G or 9A/8G; *wlaN* 9G).

** (**Bold text**) denotes the “on” status of the gene (*cj1144-45* **10A/9G** or **9A/10G**; *wlaN* **8G**).

*** no data could be obtained for *C. jejuni* 421 isolated from chicken.

**** *C. jejuni* 11168-GS does not colonise chicks.

Analysis of the on/off status of *cj1144-45* and *wlaN* genes following a 5 day colonisation in chickens, and an overnight passage in co-culture with CaCo-2 cells, showed that only *C. jejuni* strains 11168-GS, 224 and 331 changed the lengths of *cj1144-45* and *wlaN* homopolymeric tracts, whereas strains *C. jejuni* 11168-O, 421 and 520 showed no change (Table 1). *C. jejuni* 224 was shown to switch “off” both of its *cj1144-45* and *wlaN* genes following passage in chickens (changing the lengths of polynucleotide tracts from 10A/9G to 10A/10G, and 8G to 9G respectively), while no effect was observed after co-culture with CaCo-2 cells. *C. jejuni* 331 was found to switch “off” its *wlaN* gene following passage in chickens and CaCo-2 co-culture (changing the tract length from 8G to 9G) but no change was observed in the polynucleotide tracts of *cj1144-45* after either passages where it remained switched off. *C. jejuni* 11168-GS was found to change the *cj1144-45* homopolymeric G-tract length from 8 to 9 guanine residues following co-culture with CaCo-2 cells. This however, had no effect on the translation of the mRNA sequence, as the reading frame remained interrupted. Neither passaging in chickens or co-culture of CaCo-2 cells altered the on/off state of the *cj1144-45* and *wlaN* genes in *C. jejuni* 11168-GS.

### Electrophoretic Analysis of *C. jejuni* LOS

**Figure 2:**
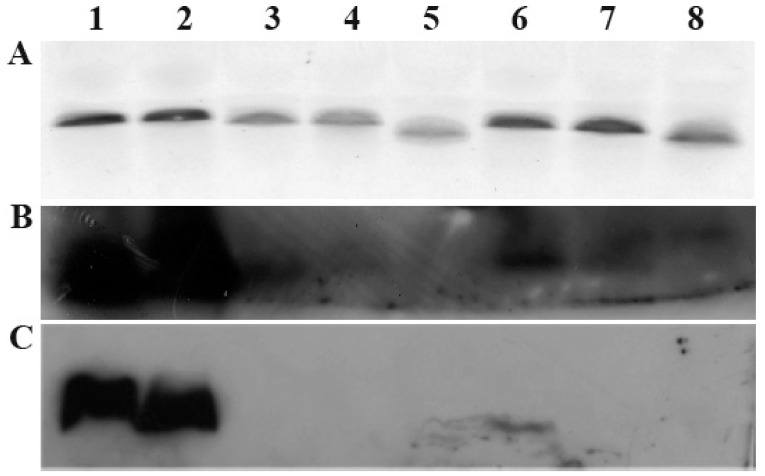
Silver stain (A), CTB blot (B) and PNA blot (C) of resolved LOS from *C. jejuni* isolates. Lane 1: *C. jejuni* 11168-O, lane 2*: C. jejuni* 224, lane 3: *C. jejuni* 331, lane 4: *C. jejuni* 421, lane 5: *C. jejuni* 351, lane 6: *C. jejuni* 375, lane 7: *C. jejuni* 520, lane 8: *C. jejuni* 81–176.

Following typing of the LOS biosynthesis clusters of the *C. jejuni* isolates 224, 331, 351, 375, 421 and 520, their LOS was analysed and compared to that of the paradigm *C. jejuni* strains 11168-O and 81–176. Firstly, the LOS forms were analysed by comparing their relative molecular weights and the silver stained banding patterns following Tricine SDS-PAGE (Figure 2A). It is important to note that in order to reduce the heterogeneity of the LOS forms produced, all bacteria were grown at 37°C. Figure 2A shows the inter- and intra-strain diversity of *C. jejuni* LOS phenotypes and further suggests that the LOS biosynthesis cluster types are not indicative of the final LOS structure. For example: class R *C. jejuni* strain 224 (lane 2) was shown to produce LOS of a similar molecular weight to the paradigm *C. jejuni* strain 11168-O LOS (lane 1), which is known to display GM_1_-like ganglioside mimicry. *C. jejuni* strains 331 (class C, lane 3), 421 (class C, lane 4) and 375 (lane 6) were shown to produce LOS of similar molecular weight, slightly lower than that of *C. jejuni* 11168-O. Interestingly, the LOS of *C. jejuni* 520, also class C, (lane 7) was shown to resolve at a lower molecular weight than that of *C. jejuni* 11168-O (lane 1) and higher than that of *C. jejuni* 81–176 (lane 8).

### Lectin Blot Analysis of *C. jejuni* LOS

In order to further describe the terminal LOS epitopes associated with ganglioside molecular mimicry and to further correlate the terminal LOS structures with LOS biosynthesis cluster types, Tricine-SDS PAGE resolved *C. jejuni* 224 (cluster type R), 331 (cluster type C), 351 (unknown cluster type), 375 (unknown cluster type), 421 (cluster type C) and 520 (cluster type C) LOS were transferred onto PVDF membrane and probed with CTB lectin (cholera toxin subunit B, CTB – differentially binds LOS with GM_1−3_-like structures) (Figure 2B) and PNA lectin from *Arachis hypogaea* (PNA – binds LOS with Galβ1,3GalNAc – terminal structures mimicking GM_1_ gangliosides) (Figure 2C). *C. jejuni* 11168-O (lane 1) and 224 LOS (lane 2) were shown to bind both, CTB and PNA, as expected of structures that mimic GM_1_-like gangliosides. *C. jejuni* 375 (lane 6) and 520 (lane 7) on the other hand, showed much weaker binding to CTB than *C. jejuni* 11168-O (lane 1) and 224 (lane 2), indicating that their LOS is likely to be lacking terminal structures such as galactose or sialic acid, which are required for strong binding to CTB. *C. jejuni* strains 375 (lane 6) and 520 (lane 7) LOS did not bind to PNA, suggesting that they do not have terminal Galβ1,3GalNAc - a moiety distinctive of G(M-Q)_1_-like structures. *C. jejuni* isolates 331 (lane 3) and 421 LOS (lane 4) bound CTB, however with much weaker binding than that of *C. jejuni* 11168-O (lane 1) and 224 (lane 2), which is consistent with the fact that their LOS is missing terminal structures such as terminal galactose or sialic acid. No binding to PNA by *C. jejuni* 331 (lane 3) and 421 (lane 4) LOS was observed, again suggesting that these LOS molecules do not have a terminal Galβ1,3GalNAc moiety. The clinical isolate *C. jejuni* 351 LOS (lane 5) exhibited no binding to either CTB or PNA indicating that it does not have a terminal Galβ1,3GalNAc moiety and hence does not mimic GM_1_-like gangliosides. *C. jejuni* 81–176 control LOS (lane 8), known to exhibit GM_2_∼GM_3_-like ganglioside mimicry, did not bind PNA and only weakly bound CTB, confirming the absence of a terminal galactose typical of a GM_2_-like structure.

### Lectin Array Analysis of *C. jejuni* Isolates LOS

Lectin blotting and silver stain were initially used for the identification of ganglioside mimicry, molecular weight determination and partial structural characterisation of *C. jejuni* LOS [14], [17]. Although it is possible to identify heterogeneity of the LOS sample, the data provided by these techniques is limited in the scope of structures characterised [17]. In order to better characterise the molecular mimicries and terminal structures of LOS isolated from *C. jejuni* strains and to correlate them with the LOS biosynthesis cluster types of these isolates, LOS samples were analysed on a lectin array. As previously described [19] the lectin array consisted of 15 lectins (ABA, ConA, DBA, DSA, ECA, JAC, LFA, MAA, MPA, PNA, SJA, SNA, UEAI, VAA and WGA) and 2 antibodies (anti-GM_1_ and anti-GM_2_ IgG) that were selected based on their binding specificities to solve the *C. jejuni* LOS structures. The lectin specificities and identified terminal LOS structures are shown in Table 2. In order to further validate the lectin array assay, *C. jejuni* strains 11168-O and 81–176 were used as controls to demonstrate positive identification of known GM_1_ and GM_2_-like LOS epitopes respectively. *C. jejuni* 11168-O LOS bound to anti-GM_1_ and anti-GM_2_ antibodies as well as SJA (a subterminal GalNAc binding lectin), MPA (an α-D-Gal/Galβ1,3GalNAc binding lectin) and PNA (Galβ1,3GalNAc binding lectin) confirming that it possesses a GM_1_-like terminal structure. Furthermore, positive binding to LFA (a sialic acid binding lectin) and MAA (a Neu5Acα2,3Gal binding lectin) correctly identified the presence of an α2,3 linked *N*-acetylneuraminic acid (Neu5Ac). Strong binding to JAC (an α-D-Gal/Galβ1,3GalNAc binding lectin) and MPA (an α-D-Gal/Galβ1,3GalNAc binding lectin) by *C. jejuni* 11168-O LOS was indicative of a terminal galactose linked to *N*-acetylgalactosamine (GalNAc), a moiety specific to the GM_1_-like structure. *C. jejuni* 81–176 LOS strongly bound anti-GM_2_ IgG and LFA (a sialic acid binding lectin) implying that the terminal structure mimics GM_2_-like ganglioside. In a similar fashion the terminal glycan structures of the LOS for all the other *C. jejuni* strains were determined (Table 2). *C. jejuni* 224 (cluster type R) LOS was shown to have GM_1_-like ganglioside mimicry as it strongly bound to anti-GM_1_ IgG, LFA, MAA, VAA, PNA and JAC (for specific lectin binding by the LOS refer to Table 2). *C. jejuni* 331 (cluster type C) LOS was deduced to have a terminal asialo GM_1_-like ganglioside structure as it bound to PNA and JAC. *C. jejuni* 351 (unknown cluster type) LOS terminus was found to mimic asialo GM_2_-like ganglioside as it bound to anti-GM_2_ IgG, PNA, ECA and JAC. *C. jejuni* 375 (unknown cluster type) LOS terminal moiety was also shown to exhibit asialo GM_2_-like ganglioside mimicry as it bound to anti-GM_2_ IgG, PNA, DSA, SJA and JAC. Similar to *C. jejuni* 351 and 375, the LOS of *C. jejuni* 421 (cluster type C) was shown to exhibit asialo GM_2_-like ganglioside mimicry as it bound to anti-GM_2_ IgG, PNA, ECA, DSA and JAC. *C. jejuni* 520 (cluster type C) LOS bound to the majority of lectins (ABA, DBA, DSA, ECA, Jacalin, LFA, MAA, PNA, SJA, SNA, UEAI, VAA and WGA) which indicated that *C. jejuni* 520 produces a heterogenous mixture of LOS mimicking GM_1_, GM_2_, asialo GM_1_ and asialo GM_2_-like gangliosides, despite being grown at 37°C, a condition known to minimise heterogeneity [17].

**Table 2:** Lectin array results.

Lectin/Antibody	Specificity	11168-O	81–176	224	331	351	375	421	520
ABA	β-D-Gal/Gal-GalNAc-serine	–	–	–	–	–	–	–	+
Anti-GM_1_	GM_1_	+	–	+	–	–	–	–	+
Anti-GM_2_	GM_2_	+	+	–	–	+	+	+	+
ConA	α−methyl-mannopyranoside > α−D-Man > α−D-Glc	–	–	–	–	–	–	–	–
DBA	Terminal α-GalNAc	–	–	–	–	–	–	–	+
DSA	GlcNAcβ1,4GlcNAc	+	+	–	–	–	+	+	+
ECA	Terminal LacNAc>Lac>GalNAc>Gal, Galβ1,4GlcNAc	+	+	–	–	+	–	+	+
JAC	Terminal α−D-Gal/Galβ1,3GalNAc	+	+	+	+	+	+	+	+
LFA	Neu5Ac/Neu5Gc	+	+	+	–	–	–	–	+
MAA	Neu5Acα2,3Gal	+	–	+	–	–	–	–	+
MPA	α-D-Gal/Galα1,6Glc/Galβ1,3GalNAc	+	–	–	–	–	–	–	–
PNA	Terminal β-Gal/Galβ1,3GalNAc	+	+	+	+	+	+	+	+
SJA	Subterminal GalNAcβ1,6Gal/GalNAc>Gal	+	–	–	–	–	+	–	+
SNA	Neu5Acα2,6Gal/Neu5Acα2,6GalNAc/GalNAc = Lac>Gal	–	–	–	–	–	–	–	+
UEAI	Terminal α1,2Fuc/Fucα1,2Galβ1,4GlcNAc	–	–	–	–	–	–	–	+
VAA	β-D-Gal/minor specificity for Neu5Ac/Gal-GalNAc/GalNAc	–	–	+	–	+	–	–	+
WGA	Manβ1,4GlcNAcβ1,4GlcNAc>GlcNAcβ1,4GlcNAc>Neu5Ac>GalNAc	–	–	–	–	–	–	–	+
	**Proposed terminal end structure of LOS**	**GM_1_**	**GM_2_**	**GM_1_**	**asialo GM_1_**	**asialo GM_2_**	**asialo GM_2_**	**asialo GM_2_**	**Heterogenous** *

+ Significant binding above the background was observed.

− No significant binding above the background level was observed.

* Heterogeneity of LOS structures was detected in *C. jejuni* 520 sample and appears to be a mix of **GM_1_/GM_2_/asialo GM_1_/asialo GM_2_ mimicry**.

It is important to note that the PNA binding results on the lectin array (Table 2) appear contradictory to the lectin blot analysis shown in Figure 2C, again showing the limitations of the blotting method. The lectin blot results illustrate the binding of PNA to *C. jejuni* 11168-O and 224 LOS indicating that both of them have a GM_1_-like ganglioside mimicry. The LOS of all other isolates (*C. jejuni* 331, 351, 375, 421 and 520) were shown not to have GM_1_-like ganglioside mimicry and hence did not bind PNA. In contrast, the lectin array experiment has positively identified strong binding to PNA by the LOS of all strains. PNA has highest specificity for Galβ1,3GalNAc, but still has affinity for all terminal galactose containing structures. The high binding avidity of LOS to lectins, printed on the glass slide, is likely to be due to the high density immobilisation of lectin molecules on the glass slide surface, which allows interactions to be detected that could not be visualised by lectin blot analysis.

### SPR Analysis of DBA Interactions with LOS Samples Purified from *C. jejuni* 520

The binding between DBA lectin and the *C. jejuni* 520 LOS sample on the lectin array was unexpected as DBA has affinity for terminal α-GalNAc and this structure has not been reported for *C. jejuni* strains encoding Class C LOS biosynthesis clusters. To further confirm this interaction, we performed binding affinity analysis between purified *C. jejuni* 520 LOS and DBA using surface plasmon resonance, SPR, analysis (Biacore). The binding affinity was confirmed at Kd of 3 µM, while the negative control assays, featuring DBA with *C. jejuni* 11168 LOS and purified GM_1_ from bovine brain, showed interactions at Kd between 1 and 300 mM. This indicates that at least some of the heterogeneous LOS produced by *C. jejuni* 520 has terminal α-GalNAc.

### Invasion and Adherence Assays of *C. jejuni* Laboratory Strains and Clinical Isolates

**Figure 3:**
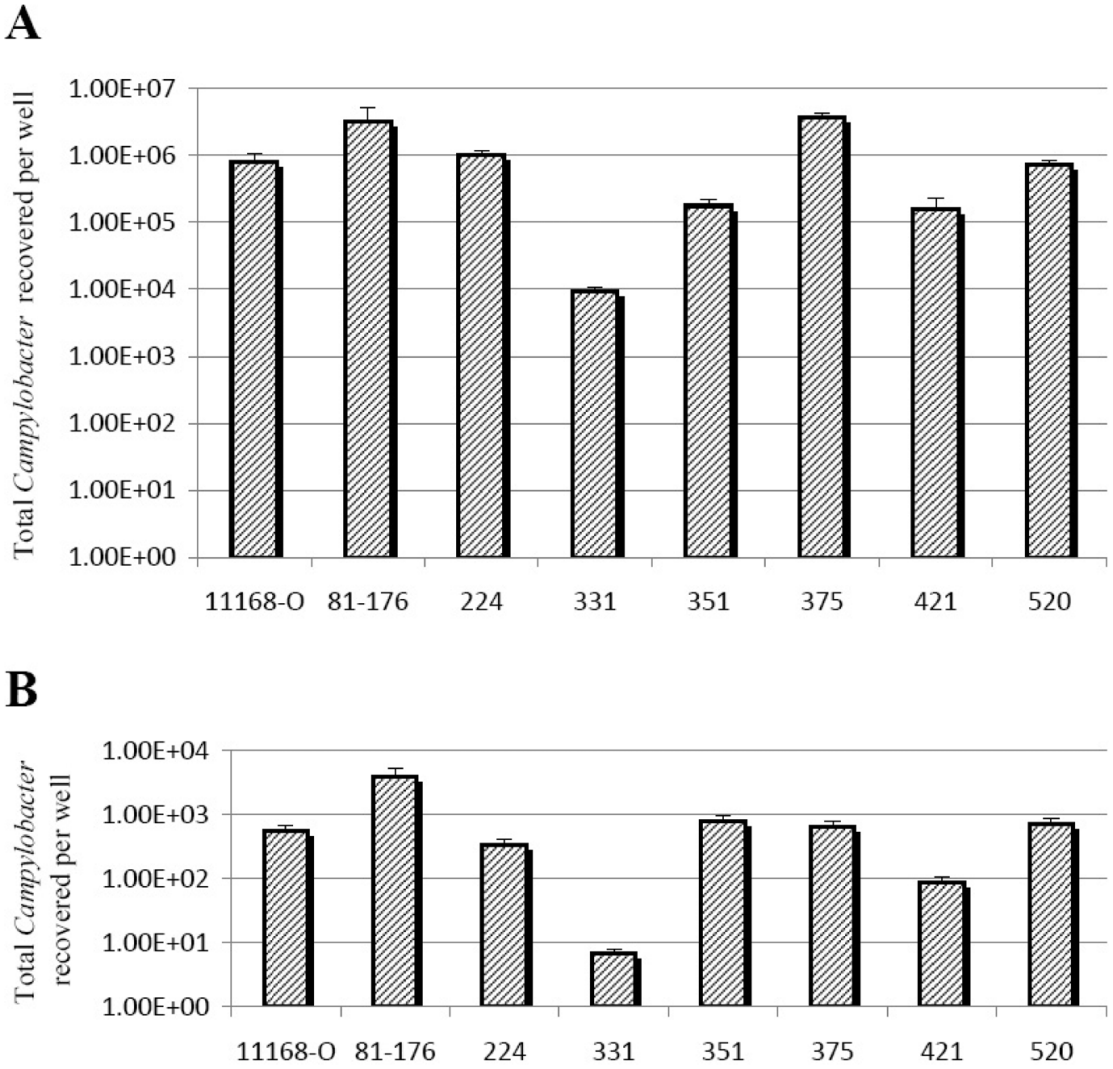
Adherence and invasion of *C. jejuni* strains. A) Adherence analysis of *C. jejuni* 11168-O, 81–176, 224, 331, 351, 375, 421 and 520 strains to CaCo-2 cells. B) Invasion analysis of *C. jejuni* 11168-O, 81–176, 224, 331, 351, 375, 421 and 520 strains into CaCo-2 cells.

LOS has previously been shown to play an important role in adherence and invasion of host cells [20], [21], [22], [23], [24], however, the correlation between the adherence/invasion profiles of *C. jejuni* strains and their LOS phenotypes, particularly with respect to sialylation is yet to be determined. In order to elucidate whether a correlation exists between the specific type of the terminal structure of the LOS and the ability of *C. jejuni* to adhere to and invade host cells, adherence and invasion assays were performed on *C. jejuni* isolates whose LOS terminal structure and LOS biosynthesis cluster type had been earlier identified. The adherence and invasive capacity of *C. jejuni* isolates 224 (cluster type R), 331 (cluster type C), 351 (unknown cluster type), 375 (unknown cluster type), 421 (cluster type C) and 520 (cluster type C) to CaCo-2 cells were assessed by comparison to *C. jejuni* paradigm strains 11168-O (cluster type C) and 81–176 (cluster type B). As shown in Figure 3, *C. jejuni* strains 11168-O, 224 and 520, which were identified as having LOS that mimicked GM_1_-like gangliosides, displayed similar levels of adherence to CaCo-2 cells, however, the invasiveness of these strains differed (*P*<0.01) (Figure 3B). *C. jejuni* isolate 331 which has a LOS that displays asialo GM_1_-like mimicry was shown to have the lowest level of adherence and invasion (1.0×10^4^ and 0.8 respectively; *P*<0.01) in comparison to the paradigm stains *C. jejuni* 11168-O and 81–176. This can be explained by the fact the *C. jejuni* 331 was isolated from chickens and is therefore not adapted to colonisation of mammalian host. *C. jejuni* strains 351 and 421 both displaying asialo GM_2_-like mimicry showed similar levels of adherence to CaCo-2 cells, but were significantly lower than that of *C. jejuni* 11168-O. Strain *C. jejuni* 421 was shown to be significantly less invasive (*P*<0.01) than *C. jejuni* 351 and *C. jejuni* 11168-O. *C. jejuni* strain 375 with an asialo GM_2_-like LOS had significantly higher levels of adherence to CaCo-2 cells (*P*<0.01) than *C. jejuni* strains (351 and 421) that have a similar LOS structure. The adherence of *C. jejuni* 375 was similar to that of the paradigm *C. jejuni* strain 81–176 that has GM_2_-like mimicry, however *C. jejuni* 375 was shown to be significantly less invasive. The adherence assays showed that there is a correlation between the molecular mimicry of the LOS and the level of adherence to the surface of CaCo-2 cells for the strains tested, demonstrated by similar levels of adherence to CaCo-2 cells by *C. jejuni* strains 11168-O, 224 and 520 displaying GM_1_-like ganglioside mimicry. Furthermore it was shown that *C. jejuni* strains that have sialylated LOS have significantly higher levels of adherence than those with asialo epitopes (*P* = 1.3×10^−4^). This is a strong indicator that sialylation of LOS promotes the attachment of *C. jejuni* to the surface of mammalian cells. Conversely, the current findings indicate that molecular mimicry by *C. jejuni* LOS does not correlate with the invasion levels observed in strains with analogous LOS structures, with strains demonstrating different levels of invasion in CaCo-2 cells (*P*>0.05).

## Discussion

Antigenic variation of bacterial surface molecules is widely acknowledged to be caused by a variety of mechanisms ranging from the regulation of gene expression to post translational modifications. The genetic basis for the generation of antigenic variation in bacterial surface molecules has been extensively studied over the last decade [14], [15], [25]. Regulation of gene expression by phase variation is documented in *Campylobacter* spp. as well as other bacteria like *Helicobacter pylori*, *Haemophilus influenzae*, *Neisseria meningitidis* and *Escherichia coli* [26], [27], [28], [29]. The mechanisms that control gene expression *via* inactivation of open reading frames without the need for an intrinsic site to introduce the mutation have also been documented in *Campylobacter* spp. [15], [30]. This study aimed to correlate the genetics encoding the synthesis of LOS in *C. jejuni* and the actual LOS structures produced by the bacteria. As described earlier, environmental factors can influence gene expression, protein folding, enzymatic activity and generally modulate cell metabolism, possibly as an intended survival/adaptation mechanism employed by the bacterium to enhance its fitness [17].

Our data showed that LOS biosynthesis cluster type C *C. jejuni* strains NCTC 11168, 421, 520 and 331 enabled production of LOS with different molecular mimicries to the expected GM_1_∼GM_2_-like mimicries, such as that of *C. jejuni* 331 which produces LOS with asialo GM_1_-like mimicry. The genetic basis for the production of the asialo GM_1_-like structure was confirmed by DNA sequence analysis of *wlaN*, *cj1144-45* and *cst* genes. The DNA sequence of *wlaN* (a β1,3 galactosyltransferase) was uninterrupted by the homopolymeric tract (8G) in strain 331, suggesting that the gene was transcribed fully, and therefore functional. This correlated with the terminal GM_1_-like Galβ1,3GalNAc moiety identified by lectin array analysis. The DNA sequence of *cj1144-45* (a putative α1,4 galactosyltransferase) on the other hand was found to be interrupted by a 10/10 double adenine/guanine tract, resulting in transcriptional inactivation of the gene. This result was consistent with the lectin array data as no terminal α-linked galactose units were identified in the LOS structure. Interestingly, DNA sequence analysis has shown that a *cst* gene homologue in the genome of *C. jejuni* 331 is a monofunctional α2,3 sialyltransferase (*cstIII*), the transcription of which was found to be terminated by an early stop codon [13]. This indicates that *C. jejuni* 331 should not be able to sialylate its LOS, which was confirmed by lectin analysis.

Furthermore, it was found that in *C. jejuni* 331 (LOS cluster type C) isolated directly from chicken intestines and CaCo-2 cells, the status of the *wlaN* was changed to “off” by the introduction of an additional ninth guanine residue into the polynucleotide tract. This is indicative that the strain preferentially produced LOS lacking terminal galactose residue during colonisation of chickens and mammalian cells.

The most remarkable was the LOS of another LOS cluster C *C. jejuni* strain 520 that was shown to have heterogeneous mix of GM_1_-like, GM_2_-like, asialo GM_1_-like and asialo GM_2_-like structures. The analysis of the gene sequences coding for the galactosyltransferases WlaN and Cj1144-45, and a sialyltransferase Cst suggests that the variable on/off profiles of these genes are likely to be involved in the production of heterogenous LOS forms. Similar to *C. jejuni* 421, the transcription of *wlaN* and the *cj1144-45* genes was terminated in *C. jejuni* 520, which suggests that an unknown galactosyltransferase is likely to play a role in facilitating the production of GM_1_-like mimicry by *C. jejuni* 520, a strain lacking genomic sequence data. The availability of additional transferases and the heterogenous nature of the LOS may provide an explanation as to why *C. jejuni* 520 LOS was able to bind ABA – a lectin with strong affinity for ABO blood antigens and Gal-GalNAc-Ser/Thr moieties with non-specific linkages. As observed for *C. jejuni* 11168-O the transcription of *cstIII* (a α2,3 sialyltransferase) in *C. jejuni* 520 was uninterrupted and therefore its LOS is decorated with sialic acid. *C. jejuni* 520 also produces non-sialylated LOS forms as its LOS bound to DBA in both array and SPR experiments – a lectin with strong affinity for terminal αGalNAc moieties which is not consistent with GM_2_-like mimicries. This is indicative of terminal αGalNAc, which is more similar to blood group antigens. To the best of our knowledge this is the first report of a terminal or subterminal αGalNAc detected in *C. jejuni* LOS. Other *C. jejuni* surface molecules have been reported to present αGalNAc as a terminal sugar, such as that seen in glycosylated proteins [32], [33]. It is not unusual for *C. jejuni* to utilise enzymes that support multiple synthesis pathways including genes shared between protein glycosylation, capsular polysaccharide and LOS biosynthesis [34], [35]. Therefore, it is possible to speculate that the LOS biosynthesis machinery of *C. jejuni* 520 may utilise an enzyme usually involved in the protein glycosylation pathway to incorporate an αGalNAc into the LOS molecule. It is important to note that *C. jejuni* 520 has an identical LOS biosynthesis cluster type to that seen in *C. jejuni* 11168. However, these two strains produce a very different range of LOS structures. This further confirms our finding that the LOS biosynthesis cluster type does not allow prediction of LOS structure. High levels of heterogeneity were observed in the LOS forms of *C. jejuni* 520, even though the strain was grown under conditions that are known to minimise heterogeneity [17].

The *C. jejuni* human isolate 224 synthesised LOS structures identical to the paradigm *C. jejuni* strain 11168-O that mimics the structure of the human GM_1_ ganglioside even though it was demonstrated that *C. jejuni* 224 has a LOS biosynthesis cluster type R, a variation of cluster type A, with the addition of *cj1144-45* (orf16). Unlike all previously tested strains, *C. jejuni* 224 was shown to have functional *cj1144-45*, *wlaN* and *cstIII* genes. The *in silico* analysis of *cj1144-45*, *wlaN* and *cstIII* DNA sequences from *C. jejuni* 224 showed that they have a high level of identity with their homologues in *C. jejuni* strains 11168 (cluster type C) and GC149 (cluster type R) respectively. This is suggestive of the functional similarity of these genes in *C. jejuni* strain 224 to characterised homologues in the paradigm strains. The genetic profile indicates that *C. jejuni* 224 should produce a Gal-GM_1_-like LOS structure, a phenotype previously not identified in the literature. The lectin array technique used in this study could not confirm the presence of a α1,4 linked terminal galactose residue in the structure of *C. jejuni* 224 LOS, however, its presence cannot be ruled out [36], [37].

Furthermore, it was observed that *C. jejuni* 224 had the “on” status of the *wlaN* gene terminated following the passage in chickens in contrast to CaCo-2 cells, where the “on” state did not change. This indicates that this bacterium preferentially produces LOS variant lacking a terminal galactose residue that promotes colonisation of an avian, but not a mammalian host. This is consistent with other studies describing that as much as 80% of *C. jejuni* isolated from chickens have transcription of the *wlaN* gene terminated [31]. However, this result is contradictory to one seen in *C. jejuni* strain 331 where the *wlaN* translation was terminated following passage in both chickens and mammalian cells. We strongly believe that differential expression of LOS genes by *C. jejuni* is strictly situational and no host specific LOS structure exists but rather it is interchanging to benefit the survival of the bacterium during the infection.

Human isolates *C. jejuni* 351 and 375 were found to produce LOS with asialo GM_2_-like mimicry as determined by lectin array analysis, however their LOS biosynthesis clusters could not be typed by the standard PCR approach, raising the possibility that some *C. jejuni* strains are able to produce LOS with structures demonstrating molecular mimicry to human gangliosides whilst having a completely different LOS biosynthesis cluster.

LOS is a primary antigenic molecule on the surface of *C. jejuni* and it plays a vital role in pathogen-host interactions. Involvement of LOS in adherence and invasion of the host cell is well documented and is of primary interest as it’s one of the major factors that determine the outcome of infection [9], [20], [21], [22], [23], [24]. Furthermore, phenotypic features of LOS such as sialylation have been shown to have a role in the induction of the host immune response through modulation of dendritic cell activation, which highlights the importance LOS in the overall outcome of the infection [8], [9], [21]. This study has documented that not all *C. jejuni* strains that have LOS biosynthesis cluster type that contains genes required for sialylation of its LOS, will produce a sialylated LOS under different conditions. We also show that there is a strong correlation between the LOS structure and the levels of adherence of *C. jejuni* to the surface of GI tract cells. *C. jejuni* strains (11168-O (C), 224 (R) and 520 (C)) which produce at least some LOS mimicking GM_1_ gangliosides have similar levels of adherence. Likewise, *C. jejuni* strains producing LOS with some asialo-GM_2_-mimicry demonstrated similar levels of adherence. On the other hand, *C. jejuni* strains (11168-O (C), 81–176 (B), 224 and 520 (C)) that have sialylated LOS showed significantly higher levels of adherence than those with asialo epitopes (*C. jejuni* 331 (C), 351 and 375 (unknown) and 421(C)). This is a strong indicator that sialylation of LOS promotes the attachment of *C. jejuni* to the surface of cells, most likely through interactions with sialoadhesin [8]. It would be interesting to further observe the role of LOS structure on adherence in both clinical and animal strains of *C. jejuni* that have a more diverse range of LOS biosynthesis clusters especially those that do not belong to classes A, B, C, M or R.

In summary, we demonstrated that the LOS biosynthesis clusters and the LOS structures produced by *C. jejuni* isolates 224, 331, 351, 375, 421 and 520 could not be correlated with the final structure of the LOS produced by the bacteria. Furthermore, structural variation in *C. jejuni* LOS was enhanced by phase variation in the genes coding for galactosyltransferases *wlaN* and *cj1144-45*. We have also shown that *C. jejuni* cells of the same strain can be decorated with more than one species of LOS and that the LOS phenotype is not static. Both of these features can be attributed to variation in the expression of LOS biosynthesis genes, which are facilitated by the response to the bacterium’s immediate environmental conditions. We have also been able to demonstrate that knowledge of the genetic composition of LOS biosynthesis cluster alone would not provide enough information to postulate the LOS structure, and that identification of a single LOS structure from a major sample fraction would not correctly depict the actual capacity of the strain to produce variant forms of LOS. It has been suggested that terminal heterogeneity of the *C. jejuni* LOS is of critical importance to interactions between the host and the infecting strain [38]. Lectin arrays enable the identification of LOS mimicry and the total capacity of each strain to produce varied LOS structures more effectively than biosynthesis cluster typing, lectin blotting or electrophoretic analysis, and more rapidly than complete structural analysis.

## Materials and Methods

### Bacterial Strains and Growth Conditions

The original isolate of *C. jejuni* NCTC 11168 (11168-O, Skirrow culture collection) was kindly supplied by D.J. Newell (Veterinary Laboratories Agency, Weybridge, UK). The human isolate, *C. jejuni* 81–176, was donated by James G. Fox (Massachusetts Institute of Technology, Cambridge, Massachusetts, USA). *C. jejuni* clinical isolates 224, 351, 375, 421, 520, and chicken isolate 331 were obtained from the Royal Melbourne Institute of Technology (Melbourne, Vic., Australia) and Griffith University (Gold Coast, Qld., Australia) culture collections. All *C. jejuni* strains were subcultured no more than once to avoid the influence of passaging. Strains were grown on blood agar, composed of Columbia agar containing 5% (v/v) defibrinated horse blood and Skirrow’s antibiotic supplement (Oxoid), under microaerobic conditions (5% O_2_, 10% CO_2_ and 85% N_2_) at 37°C for 48 h or 42°C for 24 h.

### LOS Biosynthesis Cluster Typing

The LOS biosynthesis clusters of *C. jejuni* isolates were typed by amplifying the open reading frame (orf) DNA sequences 5 (*cgtA*), 6 (*wlaN*), 7 (*cstIII*), 8 (*neuB*), 5/10 (*cgtA/neuA*), 12 (*waaV*), 14 (*cj1137*), 15 (*cj1138*) and 16 (*cj1144-45*) and overlapping gene fragments orf6-orf8 (*wlaN-neuB*), orf14–15 (*cj1137-cj1138*), orf5/10–16 (*cgtA/neuA-cj1144-45*), orf9-orf5 (*neuC-cgtA*), orf6-orf5/10 (*wlaN-cgtA/neuA*) and orf5-orf6 (*cgtA*-*wlaN*) from each of the genomes using the polymerase chain reaction (PCR). Each amplified DNA fragment combination is specific to a particular cluster type as shown in Figure 1B, hence allowing differentiation between LOS cluster types A, B, C and R as these are thought to produce LOS mimicking human gangliosides. The full gene sequences of orf5 (*cgtA*), orf8 (*neuB*), orf9 (*neuC*) and orf10 (*neuA*) were amplified, as they are involved in sialylation of LOS. Orf12 (*waaV*) present in all *C. jejuni* LOS biosynthesis cluster types was used as a positive control. The presence, as well as the on/off status of orf6 and orf16 encoding phase variable genes *wlaN* and *cj1144-45*, respectively, was identified by amplifying and analysing their full gene sequences. *C. jejuni* LOS biosynthesis cluster type C contains a unique orf14 (*cj1138)* and orf15 (*cj1138*) that were also amplified. Primer sequences for amplifying gene fragments were designed based on the published genome of *C. jejuni* NCTC 11168 (Table 3). Primers were analysed using Primer-BLAST tool (NCBI) to demonstrate that they have sufficient sequence identity with target genes from *C. jejuni* strains with LOS cluster types A, B, C, M and R. PCR reactions were optimised to contain MgCl_2_ specific to each primer set (0.5–2 mM) and were performed for 30 cycles of 10 sec of DNA melting, 30 sec of primer annealing optimised to each primer set (45–49°C) and fragment-length specific elongation time at 72°C. Amplified gene fragments were sequenced at the Australian Genome Research Facility (Brisbane, Australia) using a dideoxynucleotide approach on an AB 3730*xl* platform and the sequences analysed on 4Peaks software package.

**Table 3:** Primers used for LOS biosynthesis cluster typing.

Primer name	Primer sequence
orf 5 Forward	ATGATGTTACCTGCCATACAAAGAGG
orf 5 Reverse	CACGAATTACTTTTTCATCAAGC
orf 6 Forward	GTAGTAGATGATTGTGGTAATGATAAA
orf 6 Reverse	ATAGAATTGCTATTTACATGCTGG
orf 7 Forward	GCTTTGGTATGCGGTAATGGACCTAG
orf 7 Reverse	GGAAGTCTAATTAGATCTTTTATTAGC
orf 8 Forward	CCTTTGATAATCCCTGAAATAGGT
orf 8 Reverse	TCCTTTGCACTTATACCACCTT
orf 9 Forward	CAACTAACATGGGATGATTTTGAATG
orf 5/10 Forward	GGTGTTATAGGATATAATGATTGTACTGATGG
orf 5/10 Reverse	CCTCTGTTGTATCTATATCCAAACTAGC
orf 12 Forward	GCCACAACTTTCTATCATAATCCCGC
orf 12 Reverse	CGCCATAACTCAAACGCTCATCTATT
orf 14 Forward	GCTAGAACACCCTAAAGTGACTAA
orf 14 Reverse	TGGCACTAAATTGTAATAAATGGC
orf 15 Forward	GGTTATGTATATATGAAAACCGTAGTAGGTG
orf 15 Reverse	AATTTAATTCCGTAAAGATATTAAAAATTTC
orf 16 Forward	GGGTTGATGAAGCAAGAAATTAG
orf 16 Reverse	GTTTGGATATAGATACAACAGAGG

### LOS Preparations

Columbia blood agar-grown bacteria were harvested in 1 mL of sterile water, washed once in 1 mL of sterile water, and lysed by heating to 100°C for 5 min followed by proteinase K treatment overnight as previously described [17]. Prior to lysis, samples were adjusted for numbers of bacteria using the OD_600_ measurements of bacterial suspensions. Note that in order to reduce the heterogeneity of LOS forms present in the sample, all bacteria were grown at 37°C.

### Electrophoretic Analysis

Samples containing equal quantities of LOS were resolved on 10% (v/v) SDS-PAGE containing urea (6 M) and tricine (0.3 mM) (Tricine-SDS-PAGE) with Tricine-containing cathode buffer as previously described [17]. Following electrophoresis of LOS samples, the gels were fixed and the resolved molecules were detected using carbohydrate silver staining.

### Lectin Blotting

Tricine SDS-PAGE fractionated *C. jejuni* LOS was transferred onto Pall® PVDF membrane and was detected with horseradish peroxidase-(HRP-) conjugated cholera toxin subunit B (CTB) (3 µg mL^−1^) and with HRP-conjugated PNA (lectin from *Arachis hypogaea*) (5 µg mL^−1^) as previously described [17]. Membranes were developed using HRP Color Development Solution (Bio-Rad) or SuperSignal HRP Chemiluminescent Substrate (Thermo Scientific) according to the manufacturer’s instructions.

### Lectin Array Analysis

LOS samples were labelled with the lipophilic dye BODIPY® TR methyl ester (Invitrogen) as described by Semchenko *et al.,* [19]. LOS (5 µg) was labelled in the 0.1 µM solution the BODIPY® TR methyl ester (5 mM) in the final volume of 5 µL of milli-Q water for 10 min at room temperature prior to its direct application to printed lectin arrays. Following labelling, samples were diluted in 195 µL (1∶40) of milli-Q water and applied to the array slide separated with 25 µL gene frames (Thermo Scientific). LOS was incubated on the arrays for 10 minutes, followed by 3 washes with milli-Q water containing 0.1% Tween 20 (v/v). Controls for the BODIPY and unlabelled LOS were also applied to the array and washed in the same way. Image acquisition and data processing were performed using the ProScanArray Microarray 4-Laser Scanner and the ProScanArray imaging software ScanArray Express from PerkinElmer as described previously [16], [19]. Analysis was limited to the presence or absence of binding to lectin spots across a 1∶2 serial dilution rather than absolute binding levels. All positive binding spots were confirmed by visual inspection of the array and tested significantly above the background, which was confirmed by two-tailed unpaired T-test in Microsoft Excel. Lectin array analyses were performed a minimum of two times per LOS sample.

### Biacore Affinity Analysis

The affinity of *C. jejuni* 520 LOS to the lectin DBA was tested using surface Plasmon resonance methodology on a Biacore T100 (GE). A series S sensor chip L1 (GE) was chosen for the analysis of the lipid containing molecules. *E. coli* lipidA at 0.75 mg/mL (Sigma-Aldrich) was used as a non glycosylated control for background subtraction on flow cell 1. LOS from *C. jejuni* 520 and 11168 at 0.75 mg/mL, which were produced as described above, were immobilised on flow cells 2 and 3 respectively. A negative binding control of purified bovine GM_1_ ganglioside at 0.75 mg/mL (Sigma-Aldrich) was immobilised on flow cell 4. A standard L1 capture and sample analysis method was used from within the Biacore T100 control software. DBA was used in a 5-fold serial dilution starting from 0.1 mg/mL in PBS contain 2 mM Ca^2+^ and Mg^2+^. Immobilisation was performed for 100 seconds at a flow rate of 10 uL/minute. The DBA sample was run at a flow rate of 30 uL/minute for a total contact time of 60 seconds. A base line control was obtained using the same PBS used to dilute the DBA. The running buffer used was PBS contain 2 mM Ca^2+^ and Mg^2+^. Analysis was carried out using the Biacore T100 evaluation software surface bound affinity/kinetics tool. Affinities were determined for each of the flow cells tested, using flow cell one as a base line subtraction.

### Adherence and Invasion Assays

Adherence and invasion assays were performed using the previously described method in [39]. A confluent monolayer of CaCo-2 (ATCC) cells was inoculated across six wells with 100 µL of 1×10^8^ per mL of bacteria in each well. Infected cells were then incubated at 37°C for 1 h to allow for passive adherence and internalisation. Control assays were performed using *E. coli* in same manner (data not shown). All assays were performed at least twice. The data was transformed using natural log function in order to address the variance issue as determined by the Levene’s test. Statistical analysis of the adherence and invasion data was performed using two-tailed unpaired T-test in Microsoft Excel.

### Chicken Infection Study

Day old Ross chicks were inoculated with 1×10^8^ cfu of bacteria. Caecal content was collected from sacrificed animals following 5 days of colonisation as approved by the Griffith University Animal Ethics Committee (GU Ref No: BDD/02/10/AEC). Bacteria were extracted from the caecal content using Dynabeads® (Invitrogen) coated with whole anti-*C. jejuni* IgG [40]. The on/off status of phase variable genes *cj1144-45* and *wlaN* from the extracted bacteria was determined by PCR as described above.
